# Exploring the practicing-connections hypothesis: using gesture to support coordination of ideas in understanding a complex statistical concept

**DOI:** 10.1186/s41235-017-0085-0

**Published:** 2018-01-24

**Authors:** Ji Y. Son, Priscilla Ramos, Melissa DeWolf, William Loftus, James W. Stigler

**Affiliations:** 1 California State University, Los Angeles, 5151 State University Dr., Los Angeles, CA 90033 USA; 2 University of California, Los Angeles, Los Angeles, CA USA

**Keywords:** Gestures, Embodied cognition, Educational technology, Student learning, Statistics education, Online education, Lecture video

## Abstract

In this article, we begin to lay out a framework and approach for studying how students come to understand complex concepts in rich domains. Grounded in theories of embodied cognition, we advance the view that understanding of complex concepts requires students to practice, over time, the coordination of multiple concepts, and the connection of this system of concepts to situations in the world. Specifically, we explore the role that a teacher’s gesture might play in supporting students’ coordination of two concepts central to understanding in the domain of statistics: mean and standard deviation. In Study 1 we show that university students who have just taken a statistics course nevertheless have difficulty taking both mean and standard deviation into account when thinking about a statistical scenario. In Study 2 we show that presenting the same scenario with an accompanying gesture to represent variation significantly impacts students’ interpretation of the scenario. Finally, in Study 3 we present evidence that instructional videos on the internet fail to leverage gesture as a means of facilitating understanding of complex concepts. Taken together, these studies illustrate an approach to translating current theories of cognition into principles that can guide instructional design.

## Significance

Cognitive research on understanding has been difficult to translate into authentic learning. This may be due, in part, to the fact that understanding is not typically accomplished in a single hour-long session, the length of many psychology lab experiments. We explore three questions in the domain of statistics learning: what is understanding, how can it be assessed, and how can we design instruction that will produce more understanding in our students? We consider these three age-old questions in light of this premise: what if concepts really are embodied? How would that change assessment and teaching practices of complex ideas taught in real classrooms?

## Background

Understanding is an important goal of education. It is not enough just to learn the steps of a procedure, or be able to recall a piece of factual information. We want students to be able to think, to use knowledge they have acquired in school in new situations, for solving novel problems. The failure of our education system in achieving this goal is more and more evident, especially in the domain of mathematics. Not only do students forget much of what they learned about mathematics in K-12 education, but they show signs that the mathematics they do remember is largely disconnected from fundamental conceptual understandings of quantity, operations, and mathematical relationships. Community college students, for example, when asked a non-standard question such as, “Which is greater, a/5 or a/8 (assuming a is a positive whole number),” are reduced to guessing (Stigler, Givvin, & Thompson, 2010; Givvin, Stigler, & Thompson, 2011; Geller, Son, & Stigler, 2017).

Our recent work has focused on this problem of understanding. In particular, we are asking: what is understanding, how can it be assessed, and how can we design instruction that will produce more understanding in our students? Although these questions are not new, they have proved difficult to answer. Part of the problem may be the broad range of concepts we expect students to understand. Some concepts are simple, and are understood naturally and without much effort on the part of the learner. Vygotsky (1978) referred to these as “spontaneous concepts”. Our interest has been more in the kinds of concepts that Vygotsky called “scientific concepts”. These concepts are things you mostly learn in school, and are embedded in systems of knowledge that are handed down by culture. They often are difficult to understand and therefore require systematic instruction, as well as a great amount of motivation and effort on the part of students over long periods of time. Different processes may be involved in the understanding of different kinds of scientific concepts.

One example of such complex systems of concepts is what underlies the fields of statistics, research design, and data analysis. Although we develop intuitions about these domains through our everyday experiences (e.g., Xu & Garcia, 2008), our intuitions need to interact with more formal modes of reasoning (e.g., Kahneman & Frederick, 2002). Concepts such as variation, distribution, mean, model, sampling distribution, standard error, probability, randomness, and so on are all part of the interconnected web of concepts and procedures that make up the domain of statistics. This web of concepts does not typically arise naturally from one’s experience. These are concepts that have developed over time and are continually refined and cared for by a community of experts in the field. Students learn about these concepts in school. Through their application across a variety of situations, these concepts could give students a new way of looking at the world. Each of these separate concepts is difficult to understand in its own right (Garfield & Ahlgren, 1988; Garfield, 2003). Not only are these individual concepts abstract, but there are also many “intuitive” forms of reasoning that contradict statistical thinking (e.g., Kahneman & Tversky 1982). But even more difficult is the fact that these concepts must be understood in relation to each other, and be coordinated, as they are applied to interpret each new situation in the world.

For our purposes here, we define the process of understanding as the development of connections between a system of concepts, on one hand, and situations in the world, on the other. Hatano and Inagaki (1987) describe how understanding complex mathematical and scientific concepts takes place over time, resulting in what they call “adaptive expertise”. An adaptive expert, in their view, has developed a flexible mental model in which there is an embedded system of concepts that can be applied flexibly to both understanding and acting in novel situations. But according to Hatano and Inagaki, understanding in this deep sense is not automatic; “it requires much time and a considerable measure of conscious effort” (Hatano & Inagaki, 1987, p. 30). Most of us, in most domains, never get to that level of understanding, not because we are not capable but because we are not motivated to do so. These concepts are also difficult to study in psychology because they are not learned in a single experimental session. Statistics is a good example of this: many of our students learn how to conduct a *t* test using computer software, but fail to understand the connection of the procedure to core underlying concepts such as sampling distribution and probability. As a result, students often apply the test inappropriately, and end up drawing erroneous conclusions from data.

Deep understanding and its consequence, adaptive expertise, according to Hatano and Inagaki (1987), require the learner to spend considerable time on “comprehension activities,” which resemble in many respects what expertise researchers refer to as “deliberate practice” (Ericsson, Krampe, & Tesch-Römer, 1993). It is common to think that practice is required for the development of skills. But understanding, especially of complex scientific concepts, also requires extensive practice.

This perspective is consistent with some modern embodied theories of the human conceptual system. For example, Barsalou (2012) and colleagues propose that conceptual representations are both dynamic and situated, arising from the learners’ interactions with the world, interactions that are grounded in perception and action. Barsalou (2012) writes: “A concept is a skill for constructing idiosyncratic representations tailored to the current needs of situated action” (page 251). Embodied cognition does not merely involve the body in the moment of thinking (e.g., through gestures or use of spatial words) but posits more broadly that abstract concepts are patterned after the structures of bodily experience and are grounded in the brain’s modality specific systems (e.g., Barsalou, 2005; Damasio, 1989; Glenberg & Gallese, 2012).

In traditional cognitive theories, meaning and understanding were thought of as exclusively mental and even symbolic (Fodor, 1976; Newell, 1980), transcending modality-specific systems. In contrast, embodied cognition theories posit several ways in which concepts (even abstract concepts) maintain connections to bodily movements and interactions with the world. We will focus on three particular ways here: (1) abstract concepts are *situated*, that is, linked to situations (Glenberg & Kaschak, 2002); (2) abstract concepts are *distributed* in that they can be represented in the body and environment as a means of maintaining activation without overloading mental resources (Clark, 1998); and (3) abstract concepts are *simulation-based* in that concepts are dynamically constructed and adapted for the moment of use (Barsalou, 1999). Although this is not an exhaustive list of ways in which concepts have been written about as embodied (see Wilson, 2002), these three are relevant for our purposes.

Given that even difficult, abstract, complex concepts might be embodied (situated, distributed, and simulation-based), how would that change the way we define understanding of complex concepts in a classroom? And how would that change the way we assess and teach towards understanding of complex concepts? Under this embodied view, to understand statistical concepts requires the learner to practice the connections of the concepts to each other and to situations in the world. This process, which we see as a form of deliberate practice, involves dynamically coordinating concepts on the fly to adapt to a diversity of situations. Because of the resource-intensive nature of this process, it is often supported by embodied actions, such as gestures. We call this the “practicing-connections” hypothesis.

Drawing on what we know about deliberate practice (Ericsson et al., 1993), we would expect the practicing of connections required for understanding of complex concepts to extend over long periods of time—sometimes weeks, months, or even years. Despite common beliefs, even the most brilliant lecture about a concept such as standard deviation and its relation to other concepts is unlikely to result in flexible expertise. Under the practicing-connections view, there is no substitute for repeated practice applying the concept, and coordinating it with other concepts, in multiple situations over time. One of our goals in the work reported here was to begin exploratory research in our development of this hypothesis.

We focus on a particular example from the domain of statistics, the comparison of distributions of an outcome measure across two independent groups. In order to come to an understanding of these types of situations, statistics students must practice coordinating concepts such as the mean, standard deviation, and difference of means repeatedly across a diverse set of situations over a long period of time. These concepts are useful in a variety of situations: to summarize distributions, to consider probabilities of individual scores, to consider confidence intervals of population parameters, to compute a *t* test when asked to assess the statistical significance of a mean difference. But do students really understand what they are doing, and why? If concepts are situated and simulation-based, understanding should be measured by examining students’ ability to dynamically coordinate the concepts for the purpose at hand, which is the starting point for the current article. In our first study, we present evidence that students who have just successfully completed a college-level course in inferential statistics nevertheless are not able to coordinate fundamental statistical concepts when asked a non-standard question. With this assessment in hand, we then explore whether gesture might be one way of facilitating students’ ability to coordinate two concepts (mean and standard deviation) that must be considered simultaneously in evaluating a distribution.

Gesture has been shown in a range of studies to play a key role in the construction of complex concepts. One famous example is Piaget’s conservation of liquid quantity task. Children younger than the age of 7 or so seem unable to simultaneously consider both the height and width of a container when estimating the quantity of liquid in the container. In the classic assessment, children are presented with two identical beakers of liquid that are filled to the same level with water, and asked to confirm that the quantity of water is equivalent across the two beakers. Once agreement is secured, the experimenter pours the water from one beaker into another beaker that is both shorter and wider than the initial one, and the children are asked if the two beakers with water—one of them now tall and thin, the other short and wide—still have the same amount of water. Young children say no, generally reporting that the tall beaker has more water.

In an astonishing piece of research, Church and Goldin-Meadow (1986) found that children on the cusp of being able to correctly answer this question can be identified by their gestures when asked to explain their incorrect answers. Children in a transitional state tend to display a mismatch between their gesture and their speech during an explanation. So, for example, they will say, “The tall one has more because it’s higher,” while at the same time using gesture to represent the difference in width between the two beakers. An embodied cognition interpretation of this finding might emphasize that these children are attempting to activate and dynamically coordinate their concepts of height and width for this situation, using gesture to offload some of this cognitive work. But did the children actually have to produce the explanation themselves? What if instead of generating the explanation, children saw an experimenter present the same explanation with mismatching gestures? Would this similarly help them successfully coordinate height and width?

There is a lot of evidence that suggests that such teacher-gestures can be effective (e.g., in statistics, Rueckert, Church, Avila, & Trejo, 2017). Teacher gestures that contain information that is non-redundant with that contained in speech has been shown to benefit student learning (Singer & Goldin-Meadow, 2005). The combination of speech and deictic (or pointing) gesture is powerful (Perry, Berch, & Singleton, 1995; Valenzeno, Alibali, & Klatzky, 2003), presumably because gestures incorporate information that is perceptually present but not explicitly mentioned (Alibali & Kita, 2010). But iconic gestures that represent meaning in their form and do not reference any nearby objects are also powerful (Ping & Goldin-Meadow, 2008), perhaps because they reinforce and activate mental representations (Alibali, Bassok, Solomon, Syc, & Goldin-Meadow, 1999). Gesture and speech seem to have similar benefits in the context of video lectures (Cook, Duffy, & Fenn, 2013). Thus, we wondered if iconic gestures might be an important lever for engaging students who are trying to understand statistical concepts as presented in a brief video prompt.

In this paper, we examine whether gesture can be used (and is being used) to help students with the difficult task of coordinating statistical concepts. In the first study, we gave students a survey of some basic statistics questions to get a sense of what misconceptions currently exist among introductory statistics students in college. The results of this survey revealed that college students have fundamental misconceptions about how measures of variance within a distribution relate to the concept of statistical significance. In Study 2, we tested whether explaining a given scenario to students with gestures that highlight variance helps to alleviate misconceptions. In Study 3, we analyzed popular educational statistics videos on YouTube to understand how often these types of gestures are actually used in teaching scenarios.

## Study 1: Assessing understanding of a fundamental concept

Our goal in Study 1 was to assess what students understand about statistics right when they are just completing a college course in the subject. This study will serve as a baseline for the following studies. Although we know that most of these students can probably answer the standard problems they will face on their final exam, we asked them slightly different questions in order to assess their understanding. The assessment items we developed are part of an ongoing effort to develop a set of questions that can be used to assess whether the instructional methods used in the teaching of statistics are achieving the goals of understanding.

### Methods

#### Participants

Sixty-two undergraduate psychology students (45 female, two declined to state) at a large state university participated in the study. Students were nearing completion of an introductory inferential statistics class taught in a psychology department. Two class sections participated in this study, each taught by a different instructor. They were asked by their instructor to participate in the survey for extra credit. Even though this was a voluntary assignment, all students from one of the sections (25 of 25) and about 93% of the other section (37 of 40) participated in this study.

#### Materials and procedure

Students completed the survey online. They were presented with a scenario that could be interpreted using basic statistical concepts, and then were asked a series of questions based on the scenario. The survey took about 20 minutes to complete and included 15 multiple choice questions. In the current article we present results for the first three of these questions.

The survey started with the following scenario, titled, *The Chocolate Experiment*:

40 students participated in an experiment to find out if eating chocolate would improve test performance. Half the students were randomly assigned to the chocolate condition, in which they studied for the test while eating chocolate. The other half studied for the test without eating chocolate. As it turned out, the group that studied with chocolate scored 12 points higher than the group that had no chocolate.

After reading the scenario, students answered three multiple choice questions. The first question asked them to judge whether or not they thought there was an effect of eating chocolate on test performance. The second question asked them which pieces of additional information, selected from a list of alternatives, would be useful for determining if there was, in fact, an effect. The third question presented the same list of additional pieces of information, and asked the students to select the one that would be most useful. Exact wording of questions and response alternatives are presented in Table 1.

**Table 1:** Percentage of students selecting each answer choice for the three questions

Answer choice	Percentage of students
*Question 1.* Based on the description of the study, what would you conclude about the effect of eating chocolate on test performance?	
A. I am fairly certain there is an effect	48
B. I’m not sure—I would need more information	50
C. I’m fairly certain there is not an effect	2
*Question 2.* Which of the following additional pieces of information would be useful for determining if chocolate had an effect on test performance? (select all that apply.)	
A. The mean test scores for each group	81
B. The standard deviation of test scores within each group	74
C. A rating of how much each participant likes chocolate	16
D. A list of the exact test items that were used	27
E. More information about what kind of chocolate was used	8
*Question 3.* If you could only choose one, which of the following additional pieces of information would be most useful for determining if chocolate had an effect on test performance?	
A. The mean test scores for each group	61
B. The standard deviation of test scores within each group	24
C. A rating of how much each participant likes chocolate	6.5
D. A list of the exact test items that were used	6.5
E. More information about what kind of chocolate was used	2

### Results

Table 1 shows the distribution of responses for each of the questions by answer choice. For the first question, participants are split between being fairly certain there is an effect (48%) and needing more information (50%). The fact that nearly half of all students were “fairly certain” of an effect is striking given that, based on the scenario, they have not yet been given any information that would allow them to make inferences about statistical effects.

Participants were next presented with a list of possible pieces of additional information, and asked to select all the ones they thought would be useful for determining whether there was an effect. (Because they were free to select more than one option for question 2, the percentages reported in Table 1 do not add up to 100%.) Eighty-one percent of the students thought that the group means would be useful, and 74% thought the standard deviation would be useful. The best answer would surely be standard deviation, given that the scenario provided no information regarding variation within groups. The group means, by contrast, provide little information beyond what students might infer from the difference in means reported in the scenario. However, these two answer choices (group means and standard deviation) were each selected significantly more often than expected by chance (expected proportion = 0.5), *p*s < 0.001. The other choices were selected significantly less than would be expected by chance, *p*s < 0.001.

Finally, students were asked which piece of information they would choose if they could only choose one. Here, we see an astonishing result: nearly three times as many students said they would choose the group means (61%) compared with those who would choose the standard deviation (24%). A binomial sign test on proportion of correct responses (0.24) showed that this significantly deviated from 0.50, *p* < 0.001.

### Discussion

One of the most fundamental concepts in statistics is that within-group variation must be taken into account when judging whether there is a real difference between the means of two groups. What looks like a modest difference in means would be highly significant if the variation within groups were small. But if such variation were large, it would be less likely for the difference in means to be a significant difference. Based on the scenario we presented, it would not be possible to begin such an analysis, even informally, without some sense of what the within-group variation looks like. We would hope that students who have nearly completed a course in inferential statistics know this, and would seek out information on variation, especially when it is their statistics instructor who asked them to respond to the survey. Although students do select standard deviation as a desirable piece of information when they are selecting multiple pieces of information, when they are asked to pick one, they focus on just knowing what the group means are.

## Study 2: Using gesture to activate the concept of variation

The students in Study 1 had nearly completed a college-level course in statistics. Yet, they still did not think to bring in the concept of within-group variation when asked to evaluate a mean difference between two groups. Just as young children get focused on the height of two liquids and have difficulty taking width into account, perhaps these college students similarly get stuck on the group means as the main indicator of a group difference. Can gestures cue students to activate and coordinate the correct concepts for this situation? In Study 2, we investigated whether gesturing the concept of variation, while at the same time verbally describing a difference in means, might support students’ inclination to coordinate both mean and variation in their thinking. We did not ask participants to gesture, but instead assessed the effect of an experimenter’s gesturing on their responses to our questions.

### Methods

#### Participants

A total of 100 students (53 female) from the same large state university participated in the study. Like before, all were in the final week of a college-level statistics course; none had participated in Study 1. Students were recruited from four courses: (1) a psychology research methods course (there was some review of statistics in this course and two of the prerequisites were statistics courses); (2) a biological modeling course (this was an upper division advanced statistics course with a prerequisite statistics course; (3) a biological statistics course (covered both descriptive and inferential statistics); and (4) a statistics course taught in the school of education that covered both descriptive and inferential statistics. The survey software (Qualtrics, 2017) randomly assigned students to one of two gesture conditions: the Centrality condition (n = 47) or the Variability condition (n = 53). No students who started the study failed to complete it.

#### Materials and procedure

Students were emailed a link that took them to an online survey. As in the first study, the survey started with a presentation of the Chocolate Experiment. However, this time the experiment and its results were presented by an experimenter on video, not in writing. We constructed two versions of the video (they can be seen at http://tinyurl.com/variationgestureexperiment). The spoken words were identical across the two videos, but there was a small difference in the gestures accompanying the speech. The online survey software randomly assigned each participant to see one of the two videos.

In both conditions (i.e., on both videos), the experimenter said:

Whenever people participate in a psychology experiment, there is a distribution of scores. Some people do well, some people don’t do well, and everything in between. Forty students participated in an experiment to find out if eating chocolate would improve test performance. Half the students were randomly assigned to the chocolate condition, in which they studied for the test while eating chocolate. The other half studied for the test without eating chocolate. As it turned out, the group that studied with chocolate scored 12 points higher than the group that had no chocolate.

**Fig. 1:**
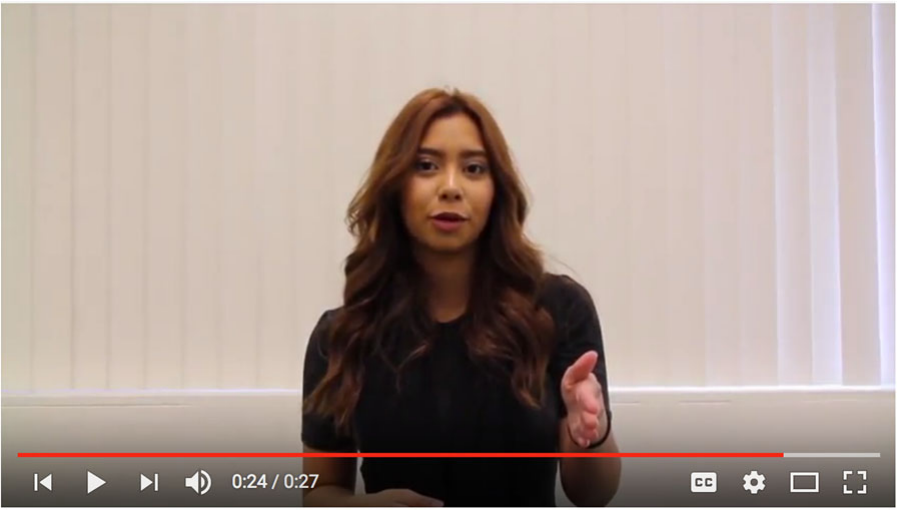
Gesture used in the centrality condition. The left hand was used to show the mean of the group that studied with chocolate, the right hand, without chocolate. Only one hand was raised at a time. Gestures matched the words, representing the difference in the two means

The only difference between the two videos was in the gestures that accompanied the last sentence, “As it turned out, the group that studied with chocolate scored 12 points higher than the group that had no chocolate.” In the Centrality condition, the gestures matched the speech in that they represented the mean difference between the groups that ate chocolate or not (Fig. 1).

**Fig. 2:**
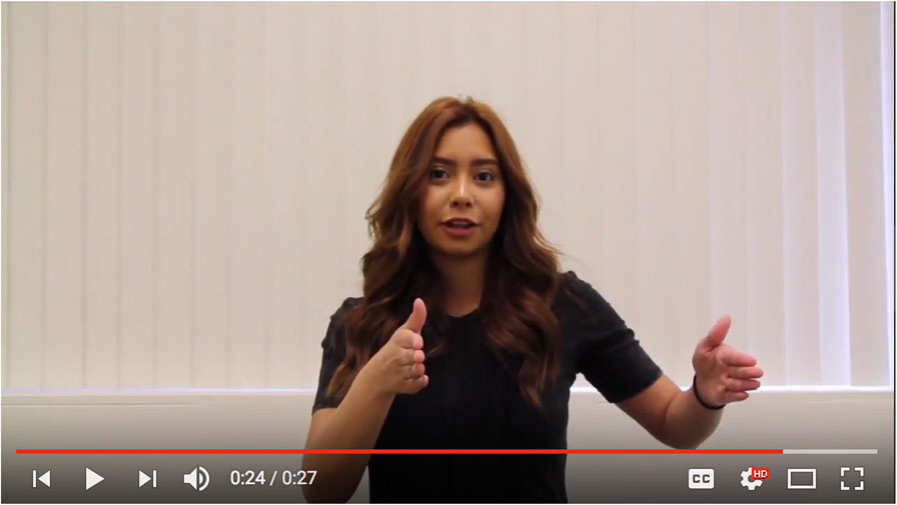
Gesture used in the variability condition. Both hands were used to represent the mean and variability of each group. In this picture the experimenter represents test performance of the group that ate chocolate. She shifts both hands to the left to represent the no chocolate group

In the Variability condition, the gestures added new information to what was contained in speech, representing not only the mean difference in test scores between the two groups, but also the fact that there was variability within each group (Fig. 2). The difference between the two groups was only in these gestures, emphasizing centrality and variability. There were no other gestures depicted in the video. After students watched the video they were asked to answer the same three questions used in Study 1, above.

### Results

The results are presented in Table 2, following the same format used to report the results in Study 1.

**Table 2:** Percentage of students selecting each answer choice for the three questions for Centrality vs. Variability gesture condition

Answer choice	Percentage students by condition	
	Centrality (n = 47)	Variability (n = 53)
*Question 1.* Based on the description of the study, what would you conclude about the effect of eating chocolate on test performance?		
A. I am fairly certain there is an effect	49	30
B. I’m not sure—I would need more information	47	60
C. I’m fairly certain there is not an effect	4	11
*Question 2.* Which of the following additional pieces of information would be useful for determining if chocolate had an effect on test performance? (select all that apply.)		
A. The mean test scores for each group	74	70
B. The standard deviation of test scores within each group	66	62
C. A rating of how much each participant likes chocolate	40	23
D. A list of the exact test items that were used	21	36
E. More information about what kind of chocolate was used	15	19
*Question 3.* If you could only choose one, which of the following additional pieces of information would be most useful for determining if chocolate had an effect on test performance?		
A. The mean test scores for each group	60	36
B. The standard deviation of test scores within each group	21	40
C. A rating of how much each participant likes chocolate	11	13
D. A list of the exact test items that were used	2	6
E. More information about what kind of chocolate was used	6	4

On question 1, the responses of students in the Centrality condition reflect a similar pattern of response as in Study 1, roughly split between being certain of an effect and needing more information. In the Variability condition we saw a different pattern, with twice as many students saying they would need more information as saying they were fairly certain of an effect. Defining “correct” as “need more information”, students’ correct and incorrect responses were subjected to chi-square test of homogeneity; this did not reveal a statistically significant difference (*Χ*^*2*^(1, N = 100) = 1.54, *p* = 0.21).

When asked in question 2 which additional pieces of information would be helpful in determining if there was an effect, students in the Centrality and Variability conditions showed a similar pattern of responses. In particular, the likelihood of selecting standard deviation as useful did not differ across the two conditions (*Χ*^*2*^(1, N = 100) = 0.2, *p* = 0.65). This result is difficult to interpret because most students (63 out of 100) chose more than one piece of additional information.

When asked more specifically in question 3 which *single* piece of additional information they would want to have if they could only have one, students in the Variability condition were twice as likely as those in the Centrality condition to choose standard deviation. This difference between the Centrality and Variability condition was statistically significant (*Χ*^*2*^(1, N = 100) = 4.59, *p* = 0.032). The distribution of responses in the Centrality and Variability conditions were not significantly different from Study 1 (*Χ*^*2*^(1, N = 162) = 5.45, *p* = 0.07).

### Discussion

Simultaneous consideration of both mean and standard deviation is a critical component of statistical reasoning, but is, apparently, a difficult thing to achieve. Yet, simply exposing students to a gestural representation of variability appears to have had an effect on their responses to our questions. Why does gesture help? The particular iconic gesture employed here illustrates the concept of distribution in an analog way. Such an analog representation may be missing in students’ default interpretation of the word “distribution” (see Singer, Radinsky, & Goldman 2008 for another case of gesture illustrating words through imagery). A spatial representation of distribution represents both the center and spread of the distribution simultaneously. Perhaps the gesture supported students’ thinking so that they could consider both variation and center of the distribution at the same time. Using gesture may have helped students either remember, attentionally highlight, or consider multiple aspects of distributions for the purpose of considering this situation.

## Study 3: The prevalence of gesture in instructional video

In Study 2, even a very brief exposure to gestures on video can shift students’ thinking. Given how frequently students search YouTube for help understanding mathematics and statistics, are the videos they find likely to leverage gesture in their explanations? Would the gestures found in these videos be iconic gestures of the sort used in Study 2? Some of the most well-known instructional videos, such as those produced by Khan Academy, do not include gesture or even hands at all. In Study 3, we examined more broadly the frequency with which instructional videos on YouTube—videos commonly accessed by students—contained gestures.

### Methods

#### Sample of videos

We typed in “standard deviation” (with quotes) into YouTube’s search field and selected for study the first 100 videos that came up in the search. The average view count of the videos was 84,718 (SD = 151,757; min–max, 291–1,091,739).

#### Coding of videos

All videos were coded by a team of trained coders. Each video was coded, first, to see if hands were visible, the face was visible, or both were visible. Those videos in which hands were visible were further coded as to how the hands were used.

Use of hands was coded based on Goldin-Meadow and Feldman’s (1975) guidelines for gesture identification. According to these guidelines, gestures should, first, be distinguished from actions. A gesture does not effect change in the environment except through its communication of meaning, so that circling an important part of an equation with a finger would be a gesture, but drawing a circle around part of an equation with a marker would be an action. However, circling part of an equation with a capped marker would be considered a gesture, as it is not effecting a change (there is no circle that remains in the environment after the gesture).

**Fig. 3:**
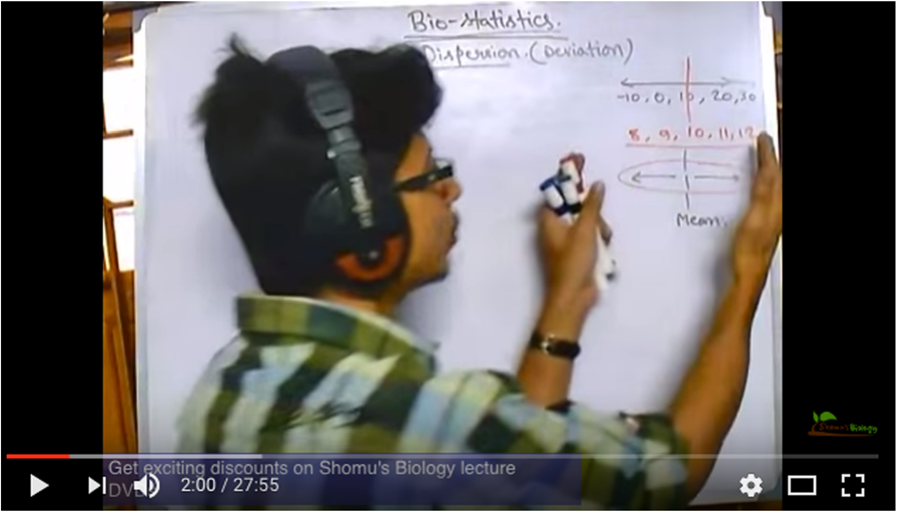
Example of iconic gesture where speaker is indicating dispersion by using the space between two hands

We coded videos for the presence or absence of three types of gestures: iconic gestures, the sort that were employed in Study 2; and two types of non-iconic gestures (deictic and other). *Iconic* gestures were defined as those whose meaning is independent of the environment, dependent only on the form of the gesture itself. Iconic gestures included “tracing” out the shape of a probability distribution with the hand, using two hands to indicate the extent of variation on the x-axis, or reaching out and grabbing an imaginary object to indicate sampling from a population (see Fig. 3 for an example). If you removed these gestures from their context, some of the original meaning would still be apparent.

**Fig. 4:**
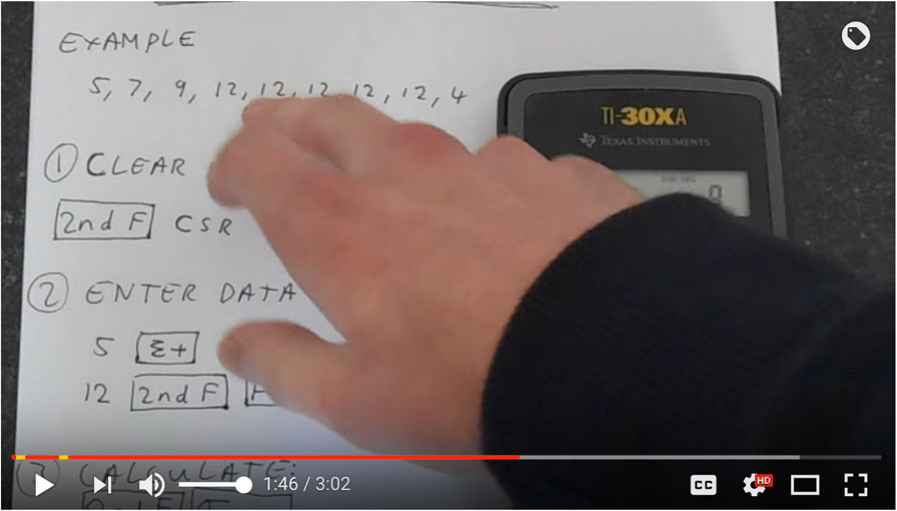
Example of deictic gesture where speaker is pointing to data points with a finger

Videos were also examined for the presence or absence of non-iconic gestures such as *deictic* gestures. These are gestures whose meaning is entirely dependent on the context of the gesture, with no meaning carried by the form of the gesture itself. A few examples of this type of gesture include pointing to part of an equation, using a finger to underline an important definition, and gesturing to a written word with an open hand (see Fig. 4 for an example). In each of these cases the meaning of the gesture is entirely dependent on what is being gestured to and how it relates to what is being said at the time. In contrast to iconic gestures, if deictic gestures are removed from their context, the original meaning is lost completely. Although iconic gestures also can reference space and other parts of the context (e.g., indicating a curve present in an image by curving the hands), iconic gestures are distinct in that their form conveys meaning even without context. Deictic gestures do not convey any meaning in their form alone.

The last type of non-iconic gesture we coded for was a catch-all category called *other.* These included beat gestures, which emphasize the cadence of spoken words, and emblems, which are gestures whose meaning is culturally defined. Moving one’s hands in time with one’s speech would be a beat gesture and giving a thumbs-up would be an emblem. These types of gestures were coded as *other* because they are unlikely to convey any information that would assist students in understanding statistical concepts.

Each video (where hands could be seen) was coded for the presence of iconic, deictic, and other gestures. Thus, a video could include all three types of gesture or only a subset of these gestures. A subset of videos was coded by five independent coders. Coders agreed on 96% of judgments. For the coding of most variables (e.g., face visible, hands visible, action, gesture, deictic gesture, iconic gesture), there was perfect agreement between five raters on five videos. Only for the coding of other was there some disagreement (*κ* = 0.19, *p* = 0.19).

### Results

**Fig. 5:**
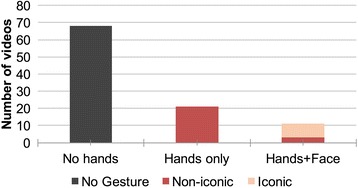
Number of videos in which hands and faces were seen and the different types of gestures that were used

The results of the coding are summarized in Fig. 5. Hands were visible in 32% of the videos (i.e., in 32 of the 100 videos sampled). Both hands and faces were visible in 11 of these videos, and only hands were visible in the other 21. Thirty-one of the 32 videos with hands included some gesture. So, instructors do gesture when talking about standard deviation, and videos that capture their hands also capture their gestures.

When gestures did appear, the most common ones by far were non-iconic (a combination of deictic and other). In the 21 videos in which only hands were visible, all of the gestures were non-iconic. The framing of these videos were typically hands writing on a surface or using a calculator. In 18 of the 21 videos with only hands, only one hand did the gesturing. The other hand was occupied (e.g., holding the paper down or grasping the calculator). Only in videos where both hands and face were visible (typically the frame included the upper body of the instructor) did we observe any iconic gestures. Eight of these 11 videos included iconic gestures, which means that only eight out of our sample of 100 videos included iconic gestures. Descriptions of the iconic gestures observed in these videos are included in Appendix.

### Discussion

Although there have been attempts in the research community to examine the effect of video-based instruction with and without gesture (e.g., Koumoutsakis, et al., 2016; Rueckert et al., 2017), most popularly accessed videos in our sample do not show hands at all. Some of these video explanations have been watched more times than a professor’s explanation would be seen face-to-face in a lifetime.

This study was limited to examining the presence of gestures at all, ignoring important characteristics such as the rate of gestures and the particular meanings of gestures as they related to standard deviation. Our main results reveal that a sampling of easily accessible videos (as determined by the YouTube search algorithm) reveals few videos with iconic gestures at all. Future studies might examine only videos that contain hands to pursue important questions about the rate of gesture and the meaning and effectiveness of particular gestures.

## Discussion

When teaching complex concepts, such as those that make up the field of statistics, we hope for students to achieve some form of what Hatano and Inagaki describe as adaptive expertise (1987). In these three studies, a story emerges. There are problems with coordinating multiple basic concepts even at the end of a college statistics class (Study 1), but teachers’ iconic gesture can help students coordinate concepts (Study 2). However, in commonly accessible statistics teaching videos, we rarely observe iconic gestures (Study 3). Each of these results can lead to a separate line of research and we will discuss the specific questions that arise. However, we were compelled to put them into one series because a broader theme of how we must integrate theories about cognition in instruction emerges.

### Why don’t students coordinate mean and standard deviation?

The results of Study 1 raise an important issue: learning about mean, standard deviation, histograms, and t-tests—all concepts covered in the participating statistics courses—was not sufficient to get students to simultaneously consider both mean and standard deviation in evaluating the effectiveness of the chocolate intervention. Although it is possible that students who were motivated by extra credit may also have been doing poorly in the class, even when the whole class or almost the entire class participates (Study 1), we observe this pattern. In Study 2, particularly in the Centrality gesture condition, we found this to be the case across a variety of undergraduate statistics courses spanning three different departments (psychology, biology, and education) and five different instructors, each implementing their own approach to teaching. Based only on our data, it is not possible to say why students did not take standard deviation into account to the extent they should. But we can propose several possibilities.

One possibility is that students were never required during their formal course to relate the *concepts* of mean and standard deviation to the procedure for conducting an independent samples t-test. Most students do learn how to calculate a t-statistic and a *p* value, and in what situations these statistics can be applied. However, learning how to calculate or use a t-test does not necessarily imply a deep understanding of how the test is *related* to mean and standard deviation. We speculate that most students would have been able to tell us that they needed the mean and standard deviation in order to calculate a t-statistic. However, although the formula for the t-statistic does represent mean and standard deviation in relation to each other, *understanding* this relationship is not necessary for performing the computation. It is possible that if we want students to understand this relationship we would need to focus on it directly.

Another possibility is that students have only a limited sense of the purpose of the standard deviation. They may have some understanding of the concept itself (e.g., its definition, its formula, how to interpret it) but have a weak understanding, if any, of how standard deviation fits within the domain of statistics. The role of standard deviation (and standard error) in a t-test is to help us see a difference in means *in terms of variability*. This ratio of the size of a difference to the amount of variation enables us to judge whether a difference is statistically meaningful (i.e., significant). Could it be that students never really appreciated the problem that was solved by including a measure of spread in a t-test? Perhaps students need experiences that would highlight the function of something like standard deviation in the solution of various problems. Such experiences might include contrasting cases (Kurtz, Miao, & Gentner, 2001), “inventing” the solution (Schwartz, Chase, Oppezzo, & Chin, 2011; Schwartz & Martin, 2004), or mental or perceptual simulations (Goldstone, Landy, & Son, 2010; Wells & Gavanski, 1989). A functional (or causal) understanding may be a prerequisite for more effective coordination of these concepts in the context of examining a group difference.

Some instructors might be surprised that students are not coordinating these concepts. In fact, some instructors find it curious that we are using mean and standard deviation as a test case of complex understanding. These concepts might seem relatively simple or basic especially compared to the rest of the topics covered in an undergraduate statistics course. However, these “basic” concepts can take on different meanings as they are connected to more and more topics in the curriculum such as sampling distributions, correlation, regression, and ANOVA. The relationship of mean and standard deviation in the context of a t-test is not the same, for example, as in the context of the Central Limit Theorem, where the mean and standard deviation of populations have a predictable relationship to the means and standard deviations of the sampling distributions they can give rise to. Students might have a sense that the concepts of mean and standard deviation are fundamental because they come up repeatedly. But to understand how they relate in specific contexts may require some support, in our case, gesture.

### Why does gesture seem to activate an initially inert concept?

Even though hints and support could have been provided in any number of ways, there are several reasons why gesture might be particularly effective for students’ thinking about complex concepts. First, teachers and students often use gesture naturally when talking about math and science (e.g., Alibali & Nathan, 2012; Crowder & Newman, 1993) so it is quite possible that in face-to-face classrooms teachers would naturally use similar gestures to talk about the meaning of standard deviation (e.g., dispersion). Perhaps these gestures are naturally occurring cues in face-to-face instruction, making them effective reminders in assessment contexts. This priming function of gesture, especially when repeated over the course of a semester, may play a role in developing more robust abstract construals of concepts.

Second, if understanding of complex concepts requires dynamically coordinating multiple concepts for each new situation, it is a resource-intensive activity, cognitively speaking, and thus subject to the limitations of working memory. When students themselves gesture, this physical offloading provides a readily available way to provide more “space” to hold additional concepts as novices work out how they are connected (Goldin-Meadow, Nusbaum, Kelly, & Wagner, 2001). When instructors gesture, these complementary sources of information may enrich students’ interpretation of spoken words by engaging different modality-specific mental resources (Baddeley, 2003). In this way, abstract concepts can be grounded in more concrete terms. Also, quantitative relationships are often taught, thought of, and spoken of in spatial terms. A picture of a distribution (e.g., as histograms, scatterplots) naturally depicts the center and spread simultaneously. Perhaps gesture, and space more generally, provides an analog system for representing and thinking about such concepts simultaneously (Kita, Alibali, & Chu, 2017).

This result adds to other research demonstrating the effectiveness of gesture and embodiment in helping students learn and work with complex academic concepts (e.g., Atit, Shipley, & Tikoff, 2014; Atit, Gagnier, & Shipley, 2015; Rueckert et al., 2017). However, our focus on gestures is not to say that other forms of non-gestural support are not important.

### Why don’t instructional videos in statistics use gestures?

One basic reason instructional videos don’t use gestures is that most videos do not even include hands. Popular screen capture software (such as Camtasia) has greatly simplified the task of making lecture videos. And, popular education websites such as Khan Academy have contributed to the proliferation of videos that include only audio and screen capture. Although this software captures everything on the screen, the speaker is typically not on the screen, or if they are, they appear as a head in a small box superimposed on a slide deck. Perhaps it is simply the availability of this kind of software that has resulted in so much video without hands. A similar story might be told about the 21 videos that included only hands. These videos were similar to the screen capture videos, except instead of capturing what was on a screen these videos showed close-ups of hands writing on paper. Again, the availability of inexpensive document cameras makes it easy to produce such videos, and this may account for their popularity.

Another possibility, however, is simply a widespread belief that gestures aren’t necessary or helpful for learning. It’s not that hard, in this day and age, to video an instructor standing at a whiteboard, teaching in much the same way they would in front of a live classroom of students. Yet, such videos accounted for only 11 of the 100 videos in our YouTube study. When videos were shot this way, both faces and hands were visible. And, as noted earlier, these were the only videos in which representational gestures appeared. If people believed gesture to be important they would no doubt shoot more videos to include them.

Our hope is that research such as that reported here, along with advances in technology, will result in an increase in the number of instructional videos that include both faces and hands. One such technology—the Learning Glass (http://www.learning.glass/)—makes it possible to video instructors as they teach behind a clear glass on which they can write and project images. Videos shot in this way show a clear picture of the instructor, his or her hands and face, and interactions of the instructor with instructional materials both with actions (i.e., drawing) and gestures. If these interactions are important supports for students’ learning, instructors’ access to these new technologies might be critical for creating effective videos.

## Conclusion: Pursuing the practicing-connections hypothesis

Beyond the modest results of three small studies, the goal of this paper was to begin laying out a framework and approach for studying how students come to understand complex concepts such as those that characterize rich domains of knowledge. Although we know a great deal about what expert knowledge looks like across a wide array of conceptually rich domains (for review see Ericsson, Charness, Feltovich, & Hoffman, 2006), we do not have a lot of experimental research on the processes through which such expertise is developed.

One reason for this is that such research is hard to do given that expertise in rich domains typically develops over weeks and months, not minutes as are typically available in psychology lab experiments. Online courses, and instructional video in particular, provide a useful tool for studying the learning of complex conceptual domains such as statistics. Because instructional videos are typically watched by individual students, it is possible to conduct random-assignment experiments in the context of ongoing authentic instruction by assigning students to watch different versions of the instruction (c.f., Stigler & Givvin, 2017).

Our work thus far is guided by what we call the *practicing-connections* hypothesis. Under this framework, conceptual understanding, like skills, results from deliberate practice situating, off-loading, and simulating concepts over long periods of time. Barsalou (2012) has proposed a theory of concept formation that is consistent with this view. The practicing-connections hypothesis proposes that learning results from repeated practicing of connections among concepts, procedures, and situations. Understanding will probably develop unevenly, over time, much the way other skills do. Gesture, because it is already highly prevalent in communicating about abstract concepts (Hostetter & Alibali, 2008; McNeill, 2000) and may represent schematization of concepts (Kita et al., 2017), has a key role to play in the practicing of such connections.

Pursuing this hypothesis will require a broadening of research on conceptual change to include learning of complex concepts over longer periods of time. Although traditional cognitive psychology seeks to understand larger units by analogy to shorter laboratory experiments, we are trying to work in authentic contexts in which the complexity is fully present from the beginning. Recent evidence in STEM education has shown that course materials developed to include gesture and practicing visualization lead to improvements in reasoning and learning (e.g., Atit, Shipley, & Tikoff, 2014; Atit, Gagnier, & Shipley, 2015; Rueckert et al., 2017). For this reason, we are building an online statistics course that we can use as a research site for exploring the implications of the practicing-connections hypothesis. By putting the course online, it gives us a way to experimentally manipulate some aspects of the course and test specific hypotheses related to instruction and learning. This work is only beginning.

## Appendix

Iconic gestures seen in these videos included:

- Jabbing to different parts of space to indicate how data are dispersed (as if making points on a scatterplot)
- Bringing hands together to indicate clusters of data
  - ° Closing fingers into a small tight circle to indicate data being close together
- Spreading out two hands with space in between to indicate dispersion (like in our videos from study 2)
  - ° Indicating deviations between data and the mean by using space between both hands
  - ° Using one hand to be the mean and one hand to represent the spread of the data below the mean
  - ° Depicting dispersion dynamically by moving hands far out from center
- Showing distance by indicating space between index finger and thumb
- Chopping with hand in middle of space to show mean
- Showing “all your data” by encircling space in front of body with both hands
- Using one hand to indicate each row of an imaginary data sheet (pausing at each row)
- Closing the hand over an equation to indicate that, after computing, we will be left with one value
- When mentioning symmetry, moving hand in a vertical up and down axis to indicate vertical axis of symmetry
